# Distinct anthelmintic classes do not affect gut bacteria, and bacteria do not alter nematocidal efficacy in *Caenorhabditis elegans*

**DOI:** 10.17912/micropub.biology.002038

**Published:** 2026-07-20

**Authors:** Laura Romanelli-Cedrez, Gustavo Salinas

**Affiliations:** 1 Worm Biology Lab, Institut Pasteur de Montevideo; 2 Biociencias, Universidad de la Republica, Facultad de Quimica

## Abstract

Soil-transmitted helminth infections are widespread and can impair children's nutrition and development. Anthelmintics may interact with the intestinal microbiota; however, they should not disrupt microbes, and microbial metabolism should not reduce drug efficacy. Using *Caenorhabditis elegans* and representative Gram-positive and Gram-negative gut bacterial lineages, *Escherichia coli* and *Lactobacillus reuteri* , respectively, we tested interactions with fluopyram, ivermectin, and levamisole. None of the drugs affected either bacterial growth, and neither bacterial lineage altered nematocidal efficacy. These results help elucidate bacteria–anthelmintic–nematode interactions using tractable experimental models; their direct relevance to *in vivo* intestinal infections remains to be confirmed.

**Figure 1. Ivermenctin, fluopyram and levamisole neither affect L. reuteri nor E. coli growth, and either bacteria did not affect nematocidal efficacy.** **A-B** Bacteria growth curves in the presence and absence of nematicides. Overnight bacterial cultures of (A) *L. reuteri* and (B) *E. coli* were used to inoculate fresh media (1:100 overnight culture:medium) and bacterial growth was monitored over time by optical density (OD) at 600 nm. Nematicides were used at concentrations that killed 100% of the worms (EC _100_ ), identical growth curves were performed with vehicle (dimethyl sulfoxide) as controls. Ivermectin (IVM) was used at 1 µM, levamisole (LEV) at 100 µM and and fluopyram (FLP) at 5 µM. Representative curves for one biological replicate are shown. Three biological replicates were performed for each growth curve. **C-H** Nematocidal activity in the presence and absence of bacteria. The motility parameter refers to the movement of a population of individuals in liquid media and was measured using the tracking device WMicrotracker ONE. The system detects motility through the interference of an array of infrared light beams caused by worm movement, and the readout is expressed as counts per unit of time. The graphs show the motility of the wild-type strain (Bristol N2 ) in the presence and absence of *L. reuteri* or *E. coli* exposed to the vehicle (control) or to one of the following nematicides: (C-D) IVM at 0.2 µM, (E-F) FLP at 3.7 µM and (G-H) LEV at 10 µM. Each point represents the motility average of 4 wells (4 technical replicates), approximately 70 worms per well, measured every 5 minutes for 300 minutes. In all cases, the counts per well at different times are normalized to the counts obtained before the addition of the compound of interest or the vehicle. This normalization corrects the small differences that may exist in the number of worms per well. A representative experiment is shown from three biological replicates. The standard error of the mean is represented by dotted lines. No statistical differences (Wilcoxon test) were observed in worm motility in the presence or absence of bacteria for any nematicide.

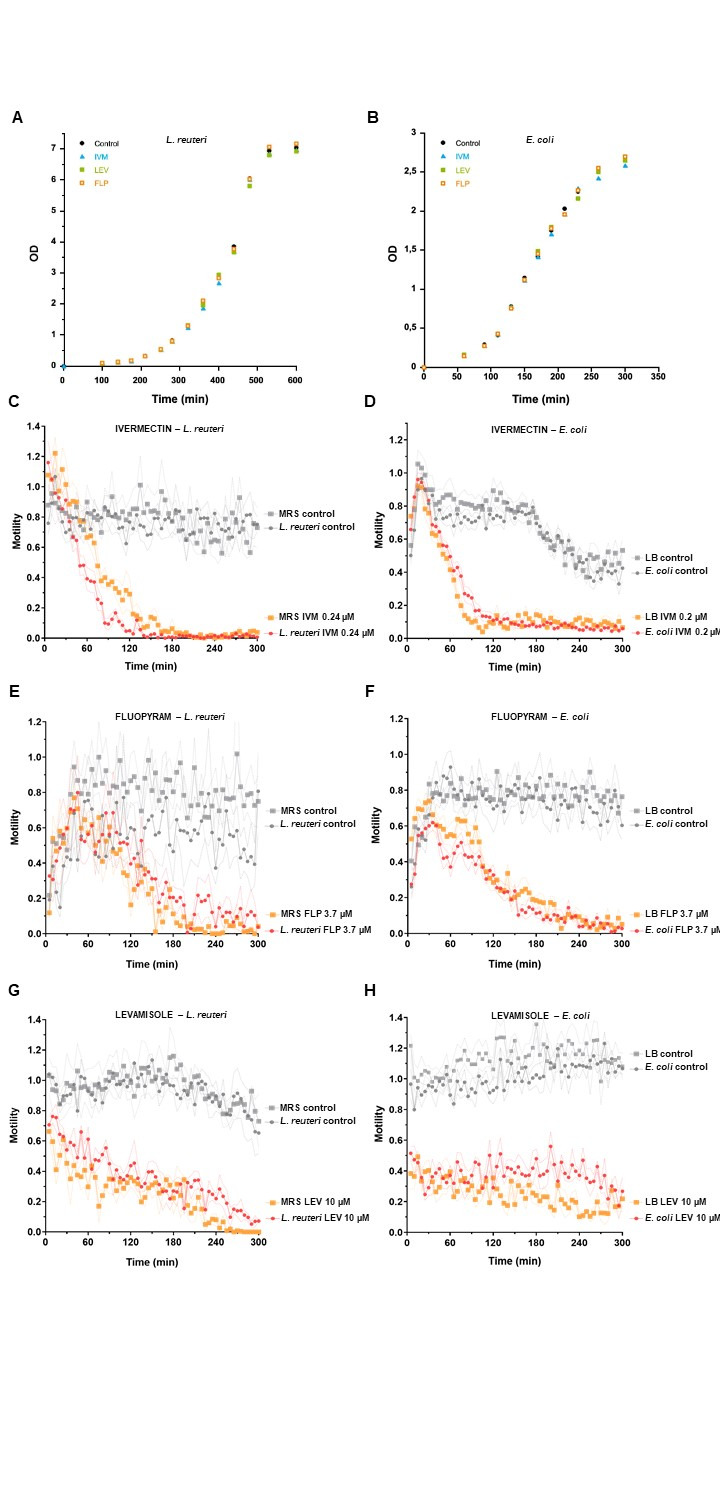



## Description

Soil-transmitted helminth (STH) infections are a major public health concern, affecting hundreds of millions of people worldwide, particularly in low income countries, and cause several neglected tropical diseases. These parasites reside in the gastrointestinal (GI) tract, leading to malnutrition, stunted growth, and cognitive impairment in children. They are caused by nematodes, such as hookworms ( *Necator americanus* and *Ancylostoma duodenale* ), *Trichuris trichiura* , *Ascaris lumbricoides* , and *Strongyloides stercoralis* , whose eggs or larvae develop in the soil before infecting humans(Bethony et al., 2006). In livestock, related gastrointestinal nematodes (GINs), which also produce soil-borne infective stages, compromise animal health and productivity. Common livestock parasites include *Haemonchus* spp, *Trichostrongylus* spp, and *Oesophagostomum* spp. Prevention includes improved sanitation, hygiene, while control involves oral drug administration with safe and effective medicines(Moser et al., 2017).

Ivermectin (IVM), a macrocyclic lactone, and levamisole (LEV), an imidazothiazole, are two widely used anthelmintics. In humans, IVM is used in *S. stercoralis* infection(Datry et al., 1994; World Health Organization, 2024), and combined with albendazole for improving efficacy against whipworm and *A. lumbricoides(Belizario et al., 2003; Palmeirim et al., 2024)* . In livestock, IVM is used as a broad spectrum anthelmintic for GIN(Peña-Espinoza et al., 2016; Yazwinski et al., 1981). LEV is often used in rotation or combination to reduce drug resistance in livestock(DeRosa et al., 2023). Both nematicides are commonly administered orally. The resistance to these nematicides has led to intensive research on discovering novel leads(Kapo et al., 2025; Shalaby, 2013). The benzamide fluopyram (FLP) is a commercial nematicide used against plant nematodes(Schleker et al., 2022), and other benzamides were later identified (notably the Wact / Wact-11 family)(Burns et al., 2015). Although FLP has shown toxic chronic effects in mammals, mostly on liver(Tinwell et al., 2014), benzamides constitute research-stage promising leads(Vairoletti et al., 2022), and thus to gain additional information about this lead is important.

An oral nematicide interacts not only with the nematode and the host, but also with the intestinal microbiota. On the one hand, the nematicide can alter the composition of the intestinal microbiome; on the other hand, the microbiota may metabolize the drug, enhancing or reducing its activity and thereby affecting its nematocidal effectiveness. A few studies have investigated how anthelmintics affect gut bacteria or whether such interactions influence drug efficacy; however, these studies are based on fecal or consortium-derived microbial communities rather than on isolated bacterial lineages(Dommann et al., 2024; Liu et al., 2023; Ma et al., 2023; Shu et al., 1991). Furthermore, there are no published studies specifically addressing gut microbiota interactions with FLP.

The free-living nematode *Caenorhabditis elegans* has long served as a key model for nematode parasitologists(Risi et al., 2024), helping to elucidate the mechanisms of action of known nematicides, investigate anthelmintic interactions, and discover new nematocidal compounds(Burns et al., 2015; Holden-Dye & Walker, 2014; Risi et al., 2024; Suárez et al., 2022). *The firmicutes Lactobacillus reuteri* and the *gamma-proteobacteria Escherichia coli* are key lineages that conform the microflora of mammalian gut. *L. reuteri* is a microorganism that actively reinforces gut health and host defense, and is also used as a probiotic(Walter, 2008), whereas *E. coli* fulfills metabolic functions and maintains the gut ecosystem balance. In this study, we examined the three-way interaction between nematicides (IVM, LEV and FLP), key microorganisms of the gut microbiota ( *L. reuteri* and *E. coli* ), and the model nematode *C. elegans* .

Because growth curves provide valuable information about how microorganisms live, adapt, and respond to their environments, we first evaluated whether IVM, LEV and FLP affect *L. reuteri* and *E. coli* growth. The microorganism growth curves were examined in the presence and absence of the three nematicides. We used the nematicides at nematocidal EC100 concentrations (1 µM, 100 µM and 5 µM, respectively). The growth curves were almost identical in the presence or absence of the nematicides; indeed, none of the phases (lag, exponential (log), stationary) were affected by the drugs at the nematicide concentrations used ( **Figure 1 A-B** ), and thus, growth of these bacteria is not affected by any of the drugs.

*We next evaluated the nematocidal effect of IVM* , LEV and FLP in the presence or absence of *E. coli* and *L. reuteri.* The results showed that these bacteria did not modify the nematocidal activity of the drugs: the EC _50_ was similar in the presence or absence of bacteria ( **Extended data** ). We then tested whether preincubation of these bacteria with *IVM* , LEV and FLP for 4 hours affect the nematocidal activity and the EC _50_ was also unaffected ( **Extended data** ).

Importantly, the decrease of worm motility over time did not change in the presence or absence of either *E. coli* or *L. reuteri* ( **Figure 1 C-H** ). These results indicate that neither activation nor inactivation of the nematicides takes place in the presence of *E. coli* or *L. reuteri.*

To sum up, our results indicate that *E. coli* and *L. reuteri* growth is unaffected by three different classes of anthelmintics (IVM, LEV and FLP), and that the nematocidal efficacy of these drugs is unaffected by these bacteria. Although the results cannot be strictly extrapolated to the treatment of parasitic nematodes living in a host's gut, this work contributes to disentangling specific tripartite interactions among bacterial lineages, nematicides, and nematodes using tractable experimental models.

## Methods

The *E. coli* OP50 strain (from the Caenorhabditis Genetic Center) was grown on LB medium at 37ºC and 210 RPM *. L. reuteri* (ATCC 23272) was grown in Man, Rogosa and Sharpe (MRS) medium at 37ºC, in the presence of 10% CO _2_ , without agitation. Overnight bacterial cultures of *E. coli* and *L. reuteri* were used to inoculate fresh media (1:100 overnight culture:medium) and bacterial growth was monitored over time by optical density (OD) at 600 nm. Nematicides were used at concentrations that killed 100% of the worms (EC _100_ ), identical growth curves were performed with vehicle (dimethyl sulfoxide) as controls. IVM was used at 1 µM, LEV at 100 µM and FLP at 5 µM. Three biological replicates were performed for each growth curve. IVM and LEV were obtained from SIGMA-ALDRICH, and FLP was provided by PROQUIMUR (Juanicó, Uruguay).

General methods for *C. elegans* culture and maintenance were performed according to reference(Sulston & Hodgkin, 1997). All experiments were performed using the wild-type Bristol N2 strain.

Nematocidal activity against worms in the presence of different bacterial strains was determined using the motility tracking device WMicrotracker™ ONE (PhylumTech, Argentina). The method used to determine worm motility is described in detail in(Simonetta & Golombek, 2007) *.* The system measures motility by detecting interruptions in an array of infrared light beams caused by worm movement. The readout is expressed as counts per unit of time (5 minutes in this study), with each count corresponding to a beam interruption produced by worms. Experiments were performed in 96 well plates, with 50-80 synchronized L4 animals per well in a final volume of 100 µL. Four wells per condition were used in each experiment (technical replicates). In all cases, counts at each time point were normalized to pre-treatment values (basal or “habituation” counts). Basal counts correspond to worm motility measured in M9 buffer (KH₂PO₄ 22 mM, Na₂HPO₄ 42 mM, NaCl 86 mM, MgSO₄ 1 mM, pH 7) alone in the absence of bacteria and nematicide. This normalization corrects minor differences in worm numbers between wells. IVM and FLP were diluted using dimethyl sulfoxide (DMSO; final concentration 2 mM). Overnight bacterial cultures were used for the assays at 1:10 dilution in M9 (overnight culture:M9). Diluted LB or MRS media (1:10 medium:M9) were used as controls.

## Data Availability

Description: EC₅₀ values for three nematicidal compounds against Caenorhabditis elegans under different experimental conditions. Assays were performed either in growth medium only (no bacteria), or in the presence of Lactobacillus reuteri (grown in MRS medium) or Escherichia coli (grown in LB medium). For each bacterial condition, assays were conducted either without pre-incubation or after pre-incubating the bacteria with the compounds for 4 hours. EC₅₀ values are expressed as mean ± SEM. n: number of biological replicates of dose-response curves. Medians were compared across medium control and bacterial groups using the Kruskal-Wallis test. No drug showed a statistically significant difference (P > 0.05).. Resource Type: Dataset. DOI: https://doi.org/10.22002/60xz4-tvm54 Description: Statistical analysis of the motility curves C to H to address one question of the reviewer.. Resource Type: Dataset. DOI: https://doi.org/10.22002/e50cj-bam56 Description: This Figure shows the statistical analysis for all the replicas of the motility curves E, G and H to address one question of the reviewer.. Resource Type: Dataset. DOI: https://doi.org/10.22002/g92hy-tkm09
